# The Synergistic Microbiological Effects of Industrial Produced Packaging Polyethylene Films Incorporated with Zinc Nanoparticles

**DOI:** 10.3390/polym12051198

**Published:** 2020-05-25

**Authors:** Szymon Mania, Mateusz Cieślik, Marcin Konzorski, Paweł Święcikowski, Andrzej Nelson, Adrianna Banach, Robert Tylingo

**Affiliations:** 1 Department of Chemistry, Technology, and Biotechnology of Food, Chemical Faculty, Gdansk University of Technology, 11/12 G. Narutowicza Str., 80-233 Gdansk, Poland; adr.banach@gmail.com (A.B.); robertt@pg.edu.pl (R.T.); 2 Department of Electrochemistry, Corrosion and Material Engineering, Chemical Faculty, Gdansk University of Technology, 11/12 G. Narutowicza Str., 80-233 Gdansk, Poland; matciesl2@student.pg.edu.pl; 3 NAN company Andrzej Nelson Małgorzata Nelson Sp. J., 35D Zajączkowo Str., 83-111 Zajączkowo, Poland; marcin.konzorski@folie-nan.pl (M.K.); pawel.swiecikowski@folie-nan.pl (P.Ś.); andrzej.nelson@folie-nan.pl (A.N.)

**Keywords:** polyethylene films, antimicrobial properties, global migration, UV barrier, zinc oxide, zinc stearate

## Abstract

Zinc compounds in polyolefin films regulate the transmission of UV-VIS radiation, affect mechanical properties and antimicrobial activity. According to hypothesis, the use of zinc- containing masterbatches in polyethylene films (PE) with different chemical nature—hydrophilic zinc oxide (ZO) and hydrophobic zinc stearate (ZS)—can cause a synergistic effect, especially due to their antimicrobial properties. PE films obtained on an industrial scale containing zinc oxide and zinc stearate masterbatches were evaluated for antimicrobial activity against *E. coli* and *S. aureus* strains. The morphology of the samples (SEM), composition (EDX), UV barrier and transparency, mechanical properties and global migration level were also determined. SEM micrographs confirmed the good dispersion of zinc additives in the PE matrix. The use of both masterbatches in one material caused a synergistic effect of antimicrobial activity against both bacterial strains. The ZO masterbatch reduced the transparency of films, increased their UV-barrier ability and improved tensile strength, while the ZS masterbatch did not significantly change the tested parameters. The global migration limit was not exceeded for any of the samples. The use of ZO and ZS masterbatch mixtures enables the design of packaging with high microbiological protection with a controlled transmission for UV and VIS radiation.

## 1. Introduction

The packaging-based industry is one of the most dynamically developing plastics processing sectors. This means that technological changes occur quickly and the requirements of processors directly translate into more and more refined properties of plastics. The global use of plastic films is growing at a rate of over 3% per year and is expected to reach 73 million tons in 2021, which is due to a greater emphasis on light and efficient packaging materials [1]. Polyolefins, mainly polyethylene (PE) and polypropylene (PP), are among the most popular polymer materials due to their relatively easy processing, low prices and functionality. The assortment of polyethylenes available for the production of flexible films includes three basic types of PE: high-density polyethylene (HDPE) with density above 0.94 g/cm^3^, low-density polyethylene (LDPE) with density range 0.915–0.939 g/cm^3^, and copolymers of ethylene with C_4_ to C_8_ ethylene-α-olefins (LLDPE) with density range 0.880–0.930 g/cm^3^. About 80% of produced LLDPE and 60% of produced LDPE are intended for film production by the most commonly used technique — extrusion blow molding. Currently, scientists are striving to develop products with increased functionality and security. Active materials are designed to extend the shelf life or keep and improve the condition of packaged products. This can be achieved by imparting properties that inhibit the development of microflora, e.g., by immobilizing bactericidal or bacteriostatic substances in the polymer structure. Antimicrobial packaging films have shown great potential to control the growth of food-borne pathogens, including *Listeria monocytogenes*, *Escherichia coli* and *Salmonella gaminara* [2]. There are known many compounds that confer antimicrobial properties to packaging materials. These include food preservatives such as benzoic acid, sorbic acid, potassium sorbate, nisin, lysozyme, and plant extracts [3]. They show great potential in inhibiting the growth of microorganisms in food products. However, standard polymer processing technologies often prevent the use of these compounds due to a lack of thermal stability that makes them incompatible with packaging material processing technologies. The development of nanotechnology has opened a new path in the search for compounds with effective antimicrobial activity not susceptible to high temperatures during processing, such as metal nanoparticles (silver, copper, zinc and gold). Metal ions have a strong antibacterial effect and also UV barrier properties [4,5,6,7]. The use of zinc compounds compared to gold or silver has one main advantage, which is a lower price. This is crucial from the point of view of industrial applications. Research on zinc oxide as an antimicrobial agent started in the early 1950s, but only in recent years there have been many reports of the antimicrobial activity of zinc oxide and zinc salts. Metal oxides, including ZnO, can be an alternative to antibiotics without causing the emergence of microorganism drug resistance [8]. Currently, zinc chloride, zinc gluconate, zinc oxide, zinc stearate and zinc sulfate are listed as a generally recognized as safe (GRAS) materials by the U.S. Food and Drug Administration [9]. Zinc oxide has a stronger antibacterial effect on microorganisms such as *Staphylococus aureus* than other metal oxides [10,11]. Zinc is also less toxic to humans compared to, e.g., silver nanoparticles [10,12]. The mechanism of action of zinc ions probably consists in the disordering of surface proteins responsible for microbial adhesion and colonization, and components such as polysaccharides and teichoic acid that protect against host defenses and environmental conditions. These components are charged macromolecules; therefore, specific interactions to disrupt their main function and location may be triggered by introducing specific groups on the surface of the nanoparticles [8,11]. Metal ions can be immobilized in polymer matrices by strong covalent bonds or by trapping in the polymer matrix. The method of application will have a direct impact on the migration parameters of active metal forms into the environment [13]. Migration parameters are often regulated by law and affect the possibility of using the material as e.g., food packaging [14]. Bumbudsanpharokea and colleagues found that the migration of zinc oxide nanoparticles from LDPE was dictated by the solubility of the polymer matrix, therefore, particular attention is needed when LDPE-ZnO nanocomposites are used for food packaging, especially acidic foods, because Zn can likely migrate, and thus their UV blocking and antimicrobial functions could be no longer effective [13]. Rokbani and others used an interesting technique of coating films with zinc oxide nanoparticles after their extrusion, thanks to which they obtained films showed considerable inhibition against *Escherichia coli* and *Staphylococcus aureus* [15]. A similar solution was proposed by Naamani et al. Using the LDPE film coating with chitosan and ZnO nanocomposites, which enabled maintaining the quality of okra (*Abelmoschus esculentus*) and inhibiting the growth of bacteria and fungi. However, this technology requires modification of the equipment used which can be a big limitation [16]. New articles have appeared on the use of zinc oxide in the creation of functional materials [13,15,17,18,19,20]. Until now, none of the papers has discussed the topic of using the antimicrobial activity of other zinc salts and the effect associated with combining zinc oxide with zinc stearate in similar applications. Obtained results present the synergistic effect of the antimicrobial action of stearate and zinc oxide nanoparticles added in the production of packaging films in the form of masterbatches. In addition, test samples were obtained on an industrial scale, which significantly substantiates the obtained results, brings the developed technologies closer to practical application and clearly shows that the problems of scaling production and mapping the properties of a product obtained on a laboratory scale have already been solved.

## 2. Materials and Methods 

### 2.1. Materials

LDPE FT5230 (923 kg/m^3^, melt flow rate 0.075 g/min, melting temperature 112 °C) and Plastomer Queo^TM^ 1001 (910 kg/m^3^, melt flow rate 0.11 g/min, melting temperature 106 °C) were purchased from Borealis (Industrivägen, Sweden), mLLDPE Exceed^TM^ 1018MA (918 kg/m^3^, melt flow rate 0.1 g/min, melting temperature 118 °C) was purchased from ExxonMobil (Port-Jérôme-sur-Seine, France), Concentrate Supporting Extrusion POLYBATCH AMF 705 HF (910 kg/m^3^, melt flow rate 0.2 g/min) and Antistatic Agent POLYBATCH VLA 55 (960 kg/m^3^, melt flow rate 2 g/min) were purchased from A. Schulman (Gdańsk, Poland). Antimicrobial agents, including ZnO nanoparticle powder with an average particle diameter of about 35–60 nm, were obtained from US Research Nanomaterials, Inc. (Houston, TX, USA) and zinc stearate nanoparticle powder with a particle diameter in the 30–50 nm range were obtained from American Elements (Lyon, France). Reagents for determination of global migration: acetic acid (99.5–99.9 % purity) and ethanol (95% *v/v*) were purchased from Avantor Performance Materials Poland S.A. (Gliwice, Poland). For microbiological tests, the following bacterial strains were used: Gram (-) *E. coli* K-12 PCM 2560 (NCTC 10538) and Gram (+) *S. aureus* PCM 2054 (ATCC 25923) from the Polish Collection of Microorganisms, Ludwik Hirszfeld Institute of Immunology and Experimental Therapy of the Polish Academy of Sciences (Wrocław, Poland). The nutrient agar was purchased from BTL Sp. z o.o. (Łódź, Poland).

### 2.2. Sample Preparation

#### 2.2.1. Granulate Preparation

The mLLDPE carrier was mixed directly with ZnO nanoparticles (50% *w/w*) or zinc stearate nanoparticles (25% *w/w*) using a low speed mixer in cycles of 4 × 1 min at a speed of 300 RPM (QZ-L300 granulate mixing mixer (Dongguan Qizheng Plastic Machinery Co., Ltd., Dongguan, China). The mixture was then extruded using a MAX 27 2-screw extruder Nurembeo with L/D=48 and a screw diameter of 27 mm (Leistritz, Nuremberg, Germany) in the temperature range of 140–160 °C. Prior to compression molding, the raw materials were dried for 10 h in a vacuum oven at 50 °C to remove moisture. The resulting masterbatch was cut into granules. The maximum mass fractions of both zinc compounds not causing processing problems were used in the granules.

#### 2.2.2. Film Preparation

The PE samples containing zinc compounds were made using Evolution Co-Extrusion line (Reifenhauser, Troisdorf, Germany). First, the appropriate amounts of LDPE, mLLDPE, CSE, plastomer, antistatic agent and mastebatches containing zinc compounds were weighed according to the data presented in Table 1. The weighed out by the precise gravimetric system components were mixed. Next, the mixture was transported to a plasticizing system equipped with bimetallic low-temperature barrier screws, in which the mixture was heated to 190 °C and homogenized. The homogeneous mixture was directed to a low-pressure blow head and formed into a sleeve with a diameter of 900–1800 mm and a film width of 500–1800 mm. The foil sleeve was cooled with a stream of air to a temperature of 40 °C and redirected to an orientation system equipped with four heated rollers, consecutively heated up to 70 °C, 65 °C, 40 °C, and 40 °C. Orientation was forced by an increase in the rotational speed of the roll by 1.5% on the first roll and 2.0% on the next three rollers relative to the speed of the sleeve advance before entering the orientation system. Then the film was wound.

### 2.3. Topography and Composition Evaluation

The samples surface morphology was characterized by scanning electron microscopy (SEM) S–3400N (Hitachi, Hyogo, Japan) using backscattered electron (BSE) detector, an accelerating voltage was 5 kV. SEM was equipped with tungsten filament. The SEM was expanded with energy dispersive X-ray spectroscopy (EDX) Ultra Dry detector (Thermo Fisher Scientific, Waltham, MA USA). The pictures of filament fragments were taken with a Nikon D7200 camera (Warsaw, Poland). 

### 2.4. UV-Barrier and Transparency

Optical property of the film samples was determined by measuring percent transmittance using UV-VIS spectrophotometer (Helios Alpha, Thermo Spectronic, Warsaw, Poland), according to Khoirunnisa and co-workers [21]. Each film sample was cut into a rectangular piece and directly place in the side of spectrophotometer cells and an empty test cell was used as the reference. Transparency of the films was tested by measuring percent transmittance at 660 nm and UV-barrier ability was tested by measuring percent transmittance at 280 nm and the average values of percent transmittance with three replicate measurements for each film sample were presented. 

### 2.5. Global Migration Analysis

Global migration was determined based on PN-EN 1186 standard [22,23,24] for following model fluids: A (distilled water), B (3% acetic acid solution), C (10% ethanol solution *v/v*) and D (95% ethanol *v/v*). A 1 dm^2^ samples were cut from the PE foils (10 × 10 cm^2^). Then, they were placed in 100 mL of model liquid in a glass, sealed vessels. The samples were placed in a laboratory chamber for 10 days at 40 °C. For the next stage of determination, evaporators with a diameter in the range of 50–90 mm and a weight of not more than 100 g were prepared. The evaporators were placed in the drying chamber at 105 °C for 30 min. Then they were transferred to a desiccator and after cooling weighed with accuracy to 0.001 g. The operation was repeated until the mass of the dish did not differ by more than 0.005g. After incubation the model liquids were evaporated to dryness in weighed evaporators, which were placed again in the dryer chamber, cooled down in desiccator and weighed with an accuracy of 0.001 g. Each test was determined in triplicate. The global migration was calculated based on the equation:

M = (m_a_ − m_b_) × 1000/S, (1)

where M is the global migration (mg/dm^2^), m_a_ is the crucible mass after evaporation of model liquid (g), m_b_ is the crucible mass before evaporation of model liquid (g) and S is the sample surface (dm^2^).

### 2.6. Mechanical Properties 

The mechanical tensile properties of the films were characterized using a universal testing machine (Instron model 5543, controlled using the “Merlin” software V 4.42., Warsaw, Poland) according to ISO 527 standard [25,26]. 

### 2.7. Antimicrobial Properties

The evaluation of antimicrobial properties of PE films was made according to the ISO 22196:2007 standard method with slight modification using *E. coli* and *S. aureus* strains [27]. Colonies of bacteria were first subcultured on triptic soya broth agar plates and incubated for 24 h at 37 °C. Microbial suspensions were prepared in test medium by adjusting the number of bacterial cells between 1.5 × 10^7^ to 5 × 10^7^ CFU/mL with spectrophotometer by measuring the absorbance at 600 nm wavelengths (optical density 0.1). The test medium was prepared by dissolving 1 g of pepton and 8.5 g of NaCl in 1000 mL of water and further sterilization. Squares with a side of 5 cm were cut from PE foil and sterilized by 95% *v/v* ethanol immersion. Next, each material was pre-wetted with water for 1 h prior to the addition of inoculum. Composites were exposed to test organisms on separate dishes by spreading 0.2 mL of prepared inoculum across the surface of the sample. Then, each square of inoculum on the surface was covered with sterile square-shaped polyethylene film 4 cm long to ensure contact of the cell suspension with the material on the surface of 16 cm^2^. Immediately after inoculation (time 0) and after 24 h incubation (37 °C), samples were placed in 10 mL of test medium and vortexed intensively for 25 s. Next ten-fold serial dilutions were plated on TSA plates and incubated for 24 h at 37 °C. After the incubation, only plates where 30 CFU to 300 CFU were counted. When no colonies were recovered the number was recorded as “10” and the number of viable counts was established (CFU/mL). We obtained the viable count of the bacteria according to the following formula:

Vc = N × D,(2)

where Vc is the bacteria concentration, in colony forming units per mL (CFU/mL), N is the average value, in colony forming units (CFU) from Petri dishes, and D is the dilution factor from the plates counted.

Antimicrobial activity on a logarithmic scale was calculated according to the formula:

R = log (B/A)(3)

where A is the average of the number of viable cells on the test sample after 24 h incubation at 37 °C (CFU/mL) and B is the average of the number of viable cells on the control sample after 24 h incubation at 37 °C (CFU/mL). A percentage reduction of bacteria/fungi on a logarithmic scale (R) equal to 1, 2, and 3 corresponds to a reduction of 90%, 99%, and 99.9%, respectively. 

### 2.8. Statistical Analyses 

The STATISTICA software (StatSoft, Inc., Tulsa, OK, USA) was used for analyses. The statistical significance was determined at *p* < 0.05. All data reported were based on the means of three replicates (*n* = 3). Experimental results were expressed as mean ± standard deviation (SD). Student’s t-test and one-way analysis of variance (ANOVA) were applied (Tukey post hoc test). The differences were considered to be statistically significant at *p* < 0.05.

## 3. Results and Discussion

The results described below apply to the production of polyethylene-based films with functional additives in the form of masterbatches containing zinc nanocompounds: zinc oxide (ZO) and/or zinc stearate (ZS). According to the available scientific literature, zinc compounds exhibit antimicrobial activity and affect the transparency of films in which they occur [21,28]. Main component of the produced films was low-density polyethylene. The next components in the decreasing percentage were linear low-density polyethylene (mLLDPE), plastomer, concentrate supporting extrusion (CSE), and antistatic agent. mLLDPE was used to produce a thinner film, with a better resistance to stress due to its higher resistance to tensile and impact than LDPE [29]. The role of plastomer was reducing crystallinity, melting point and improving flexibility during blown film processing. CSE was used as an additive supporting the process of film extrusion using LLDPE/LDPE blends with a high content of linear polyethylene, eliminating the separation of the melt polymer stream, reducing pressure in the blow head and supporting the homogenization of plastics in plasticizing system. The long-acting antistatic additive was necessary to eliminate the electrostatic charge of PE films accumulating during processing or use of the finished product.

### 3.1. Transparency and Composition

**Figure 1:**
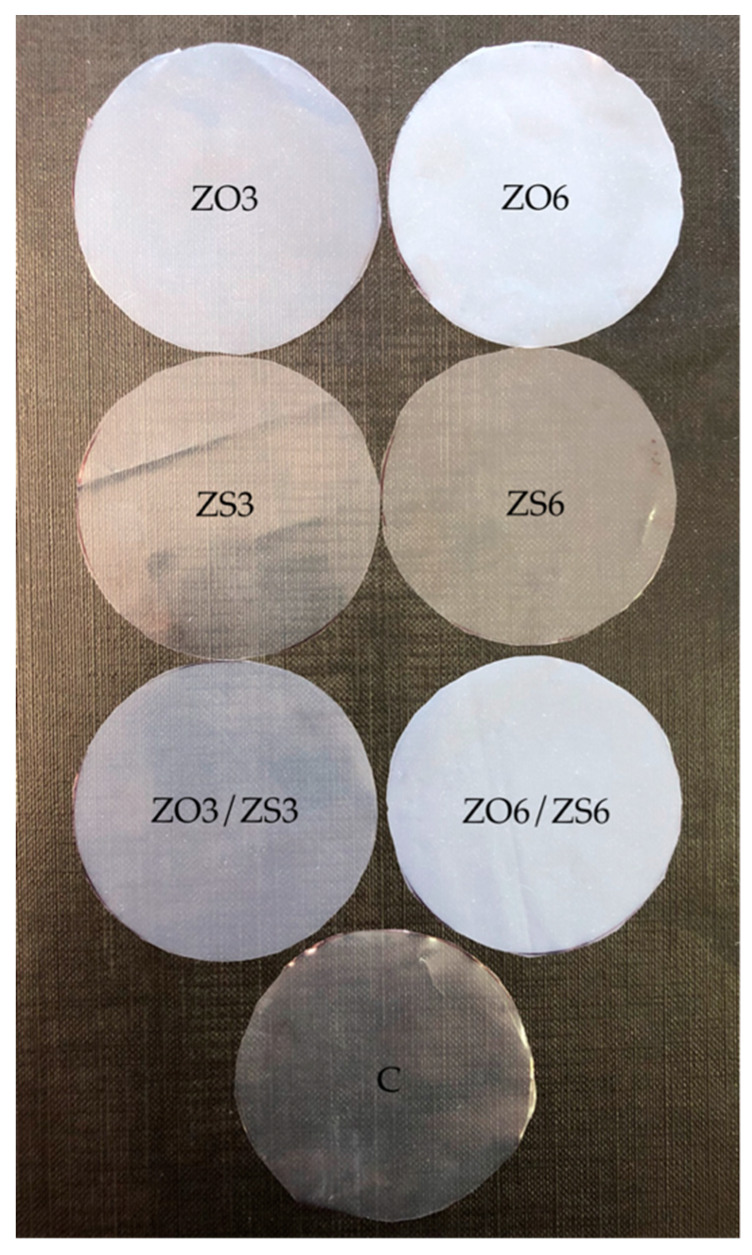
Comparison of the appearance of PE films containing a masterbatch with zinc oxide (ZO) and/or zinc stearate (ZS). (The number next to the additive symbol means its content in the sample in % *w/w*, C—control sample without zinc compounds).

Figure 1 presents photographs of samples cut from produced PE films. In visual assessment, the most transparent were samples containing masterbatch with zinc stearate in both tested concentrations and a control sample. Samples containing zinc oxide masterbatch became less transparent as the proportion of this additive increased. According to this relationship, a sample containing a mixture of both additives, each with a concentration of 3%, should be visually similar to the ZO3, which is confirmed by Figure 1. The same relationship applies to samples with 6% masterbatch additives. Visually, ZO6 and ZO6/ZS6 sample seem to be the same.

The ability of packaging films to protect against UV radiation is a desirable feature, because it allows to reduce or prevent photosensitized oxidation of fats in food, discoloration of packaged food products and leading to loss of nutrients [30]. Table 2 shows the transmittance of light at UV and visible region determined at 280 and 660 nm, respectively. Control PE film has lower transmittance values at visible region (660 nm) compared to UV region (280 nm). The use of a masterbatch additive with zinc oxide significantly reduced the transmittance of PE films in UV light, and thus increases its ability to screen radiation in this range. The UV light transmittance through ZO3 and ZO6 film was about 50% and 72% lower than in control sample, respectively. The same level of reduction in UV transmittance was observed for the ZO3/ZS3 and ZO6/ZS6 samples. Moreover, samples containing only zinc stearate additive did not significantly affect the UV transmittance. The result showed that only ZnO particles introduced into the PE film in the form of ZO masterbatch lead to higher UV shielding capacity. Zinc oxide (ZnO) is a semiconductor material with a bandwidth energy of about 3.2 eV. As a result, ZnO absorbs ultraviolet (UV) light through the electronic excitation process between the valence band and the conduction band [31]. Similar results were observed for other films containing zinc oxides [32,33].

Transparency is an important physical property of packaging films that ensures or prevents light transmission [30]. The higher the film transmittance value, the better the transparency, because more visible light (660 nm) is able to pass through the film. The obtained results indicate that the use of masterbatch with zinc stearate at both tested concentrations does not affect this visible light transparency significantly compared to the control sample (Table 2). Significant changes in visible light transmission were noted for tests of PE films with the addition of masterbatch containing zinc oxide, where for the ZO3 sample the transmittance at 660 nm was 15% lower. The ZO6 sample was completely impermeable. The similar results were obtained for samples in which masterbatch mixtures were used, which clearly shows that the transmittance for both tested wavelengths strongly depends only on the concentration of ZO masterbatch. Moreover, these results overlap with the visual assessment of the samples.

**Figure 2:**
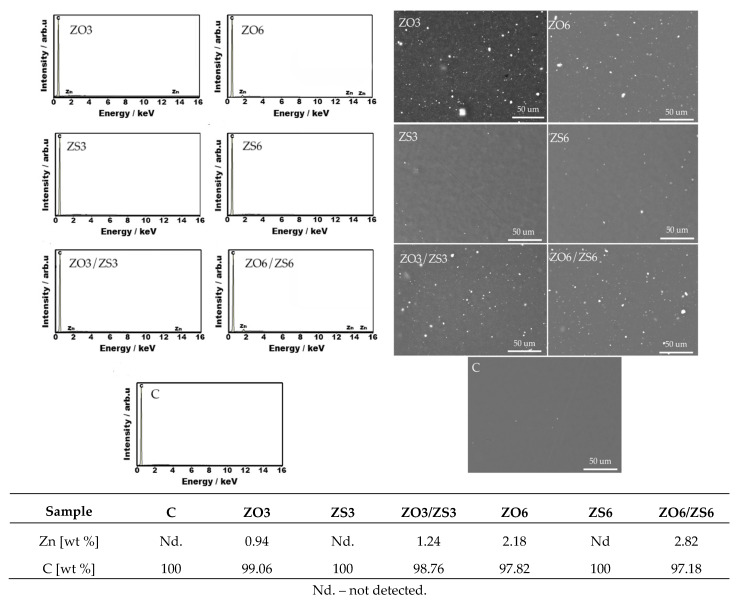
Composition of PE films containing a masterbatch with zinc oxide (ZO) and/or zinc stearate (ZS) compared with EDX spectra and SEM image of surface morphology at 500× magnification.

The presence of zinc in PE films was confirmed by micrographs obtained by scanning electron microscopy, which is visible in the form of white dots on a gray background (Figure 2). Under the shear force in the extrusion process, the zinc compounds were well distributed all over the surface and inside of polymer matrix. Larger amounts of zinc can be observed in ZO3, ZO6, and ZO3/ ZS3 and ZO6/ZS6 samples. In each of these samples a smaller amount of white-clouded clots with non-uniform distribution can be seen indicating a partial agglomeration of the filler [13]. The amount of zinc in tests ZS3 and ZS6 samples turned out to be lower than the other samples, in the form of single dots (except the control which is the correct result). When comparing micrographs with literature results, the zinc additive manufacturer’s declaration of particle sizes smaller than 100 μm seems to be correct [19]. 

Figure 2 also contains the results of the assessment of zinc content expressed as a mass percentage and calculated on the basis of EDX spectra. Taking into account the percentage share of zinc in the molecular weight of zinc oxide (80.3%) and zinc stearate (10.3%), the content of these salts in their masterbatches equaled 50% and 25%, respectively, and assuming at least 95% purity for the zinc salts used for masterbatch production, the theoretical zinc content calculated on the basis of mentioned indicators differs by up to approx. 20% from the result obtained on the basis of the EDX technique. Values lower than the theoretical were obtained for ZO3 and ZO6 tests by 18% and 5% respectively. Zinc was not detected in the stearate tests, although theoretically its content in the ZS3 and ZS6 tests should be 0.073% and 0.147%, respectively. However, in samples containing 3% and 6% of both masterbatches, the zinc content was 2% and 14% higher than the theoretical. Considering the measurement conditions (accelerating voltage), it can be suggested that zinc oxide nanoparticles may partially agglomerate in the deeper layers of the material, and the use of additional zinc stearate allows combined better dispersion of additives in the polymer matrix.

### 3.2. Global Migration 

All materials that come into contact with food must be carefully controlled to avoid the risk of contamination and to ensure compliance with safety and health requirements. Global migration is the total mass of all non-volatile substances released from the material or article, under specific test conditions, into the model liquid imitating the food [14]. There are hardly any detailed criteria for specific nanomaterials that can be used in the food industry. Even the final FDA guidelines state that it does not consider all products containing nanomaterials or the use of nanotechnology as dangerous [34,35]. When it comes to nanotechnology for food applications, the European Union has implemented the most detailed regulations. Guidelines in the form of EU Commission Regulation (No. 10/2011) dealing with plastics and other materials which come into contact with foods was implemented in 2011 [14]. According to these guidelines, the migration limit of 10 mg/dm^2^ per one-kilogram cubic packaging of food and equal to 60 mg/kg of food was set, but with the exclusion of nanoparticles. Moreover, the regulation states that the authorization to produce or use a material in bulk does not imply the authorization to use it in nanoform. Later, an amendment to this regulation for new nanoform materials, including zinc oxide and food packaging usage conditions, was laid down [36]. There is no specific information in the regulations of the European Union about the possibility of using zinc stearate in the production of plastic-based packaging. However, in Article 6 of EU Commission Regulation (No. 10/2011), in derogations for substances not included in the Union list zinc salts are mentioned, which means that if the migration limit is not exceeded for them, such compounds can be used in production. The obtained results of the determination of global migration to simulants of all types of food products under standard test conditions (40 °C, 10 days) were well below the allowable limit of 10 mg/dm^2^ (Table 3).

The migration levels were 0.041–0.1.68 mg/dm^2^ in distilled water, 0.124–1.230 mg/dm^2^ in 3% acetic acid solution, 0.0019–0.0051 mg/dm^2^ in 10% (*v/v*) ethyl alcohol water solution, and 0.0010–0.0020 mg/dm^2^ in 95% (*v/v*) ethanol. It can be observed that the increase in the amount of migrating packaging components is associated with increase in the concentration of masterbatches in PE films, in particular for the ZO masterbatch. The highest level of migration was observed in the acid model fluid, while the lowest was seen in ethanol. These results can be explained by the solubility measurements which were carried out by Bumbudsanpharoke and co-workers that showed the same tendency [13]. The solubility in any food simulant affects the stability of fillers embedded in the polymer matrix. Higher solubility allows the release of more zinc from the PE film. Previous studies reported that the dissolution of engineered ZnO nanoparticles is highly dependent on the pH of the contact media [37]. Moreover, the dissolution of ZnO nanoparticles occurs rapidly in artificial lysosomal fluids (pH 5.5) but stops in interstitial fluids (pH 7.4) [38]. Furthermore, Ozaki et al. examined the migration of Zn from food contact plastics into food simulants (distilled water, 4% acetic acid and 20% ethanol) and found that Zn migration was highest in 4% acetic acid due to a higher tendency to ionize [39]. Zinc stearate is a hydrophobic compound, insoluble in polar solvents, which is why the migration from samples containing ZS masterbatch was significantly lower than from ZO and ZO/ZS samples. Considering these references, the state of nanoparticles migrated from polymer matrices into food simulants is mainly in the ionic form due to the dissolution caused by the solvent entering the polymer through diffusion [40].

### 3.3. Mechanical Properties

Tensile strength, elongation at break and Young’s modulus were determined from stress-strain curves as shown in Table 4. The use of the ZO masterbatch significantly influenced the mechanical properties of PE films. Regardless of the concentration of this additive, the value of tensile strength increased by approx. 23%. This phenomenon is attributed to the action of the zinc oxide molecule as a compatibilizer improving the stress at break parameter due to the significant interfacial adhesion and interaction between the LDPE matrix and ZnO nanoparticles [41]. Venkatesan observed that the tensile strength increased with filler content up to 7 wt % and decreased thereafter. This discontinuity could be attributed to increased filler quantity leading to a weaker filler-matrix interface and agglomeration of filler particles, which consequently decreases the strength. This is probably because of better interfacial adhesion between the filler and the matrix by the van der Waals or induction interactions [42]. This is in line with our results.

For ZO3 and ZO6 samples, a 6% and 20% increase in Young’s modulus was also noted, respectively. Elongation at break decreased with the increase of additive concentration, regardless of what type of masterbatch was used, what may be the result of poorer dispersion of fillers associated with agglomeration of nanoparticles [41]. The addition of ZS masterbatch did not significantly affect the tensile strength of the samples. However, it increased the modulus of elasticity by 11% and 12% in the ZS3 and ZS6 samples, respectively. Tensile strength of ZO3/ZS3 and ZO6/ZS6 do not differ statistically from the values obtained for samples containing only the ZO masterbatch. Young’s modulus of samples with masterbatch mixture was higher than for samples with single masterbatch addition of the same concentration. It reached the highest value for the ZO6/ZS6 sample (by 32% higher than in the control sample). In turn, elongation at break for the same sample was the lowest, by 42% lower than in the control sample. (Table 4). Comparing our results with other literature reports, similar relationships between the increase in tensile strength, Young’s modulus and reduction of elongation at break due to the use of zinc oxide fillers can be seen. However, they are the resultant of the concentration and particle size of filler and properties of polymer matrix [19,41,42]. In summary, all the tested samples have moderate properties in terms of tensile strength (10–100 MPa) and good properties due to the ability to deform at the moment of breaking (> 50%) [43].

### 3.4. Antimicrobial Properties 

**Figure 3:**
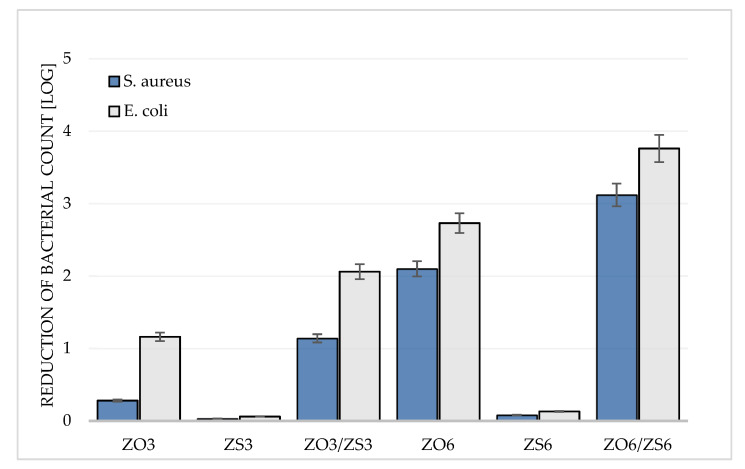
Comparison of the logarithmic reduction of bacteria cells number after incubation with PE films containing a masterbatch with zinc oxide (ZO) and/or zinc stearate (ZS) with respect to the Control sample after 24 h incubation.

To evaluate the antimicrobial properties of the obtained films ISO 22196:2007 standard method with slight modification was used [27]. This is a quantitative method, standardized according to JIS Z 2801, dedicated for measurement the antimicrobial activity of plastics [44]. The antimicrobial activity determination results were presented in the form of degree of reduction achieved after 24-hour contact of the *S. aureus* and *E. coli* inoculum with the tested materials (Figure 3). 

The obtained results confirm the ability of PE films containing zinc additives to inhibit the growth of *S. aureus* and *E. coli*, although the latter seems to be more susceptible to the antimicrobial effect of tested materials (Figure 3). A greater antimicrobial effect at the same concentration of additive can be obtained for materials containing ZO masterbatch. ZO3 samples showed a 0.28 and 1.16 logarithmic decrease in the number of *S. aureus* and *E coli* cells, which translates into a 42% and 91% reduction, respectively.

For the same concentration of ZS masterbatch addition, the logarithmic reduction was 0.03 and 0.06 (7% and 13% respectively). ZO6 samples showed a decrease in the number of *S. aureus* and *E. coli* cells by 2.10 and 2.73 in logarithmic scale (99% and 100%), while ZS6 samples again showed lower activity at the same concentration. They caused a reduction in the number of bacteria on a logarithmic scale equal 0.08 and 0.13 for *S. aureus* and *E. coli*, respectively, which means a 12% and 23% reduction (Figure 3). An important highlight is the fact that the use of both masterbatches in one PE material allows achieving a synergistic effect of antimicrobial activity. This means that the ZO3/ZS3 and ZO6/ZS6 samples inhibit the growth of microorganisms more strongly than would result from the sum of the ZO3 and ZS3 activities as well as ZO6 and ZS6 respectively (Figure 3). The literature reports that zinc oxide is an effective antimicrobial agent whose action may be based on several activities [17], including the production of reactive oxygen species due to the semiconductor properties of ZnO [45], the microbial membrane destabilization upon immediate interaction of cell walls with zinc oxide particles [46], and the antimicrobial intrinsic characteristics of Zn^2+^ ions emancipated by the ZnO particles in medium of aqua [47]. Antimicrobial effect of zinc oxide is determined by its concentration in the material and the size of its particles [48]. In case of zinc stearate particles, our research indicates activity of films containing ZS masterbatch in inhibiting growth against both bacterial strains (max. about 20%), but it is hard to speak of antimicrobial function here. The antimicrobial effect of zinc compounds is not clearly defined and is based on several mechanisms. The literature lacks information on the potential mechanism of action of zinc stearate in the creation of microbial protection in various types of polyolefin-based films. The use of zinc stearate in antimicrobial blends is mainly known for using it as a matrix for other antimicrobial substances such as silver nanoparticles [49,50]. The low activity of materials with the ZS masterbatch will seem obvious, because it results from the lower content of zinc in its salt with stearate and the concentration of zinc stearate in masterbatch. In turn, an explanation of the synergistic antimicrobial effect of both zinc salts in the ZO3/ZS3 and ZO6/ZS6 materials may underlie the mechanism involved in the destabilization of the bacterial cell membrane. Membrane potential and permeability are closely related to the bacterial sensitivity to the ionic environment. Ion homeostasis affects the multiplication, communication, metabolism and survival of bacteria [51]. Zinc ions have been reported to alter the potential of the mitochondrial membrane of rat brain cells [52,53]. First, the synergy effect probably results from the total concentration of zinc from both masterbatches, which causes greater disturbance of the cell membrane potential. When the extracellular Zn^2+^ concentration increases, as a result of the release from the surface of ZO, ZS, the bacteria transport some cations, including Zn2+, to the cell to maintain membrane potential. However, it is an inhibitor of ATP synthesis and oxidative phosphorylation in cells. P-type ATPases are the essential enzymes involved in bacterial resistance to heavy metal ions [54]. The intracellular accumulation of cations caused in this way can eventually lead to cell destruction [55]. Simultaneous cooperation of other mechanisms at the same time is not excluded. Moreover, one of the applications of zinc stearate in industry is the stabilizing, emulsifying and slipping function. The presence of ZN masterbatch can cause an increase in the amorphous structure of polymers in the area of the material surface. Within this structure, it is able to contain more water molecules, which translates into greater ion release and ultimately an increase in antimicrobial activity.

## 4. Conclusions

The research confirms that the use of the ZO masterbatch allows the design of antimicrobial packaging with limited permeability to UV and VIS radiation and slightly better mechanical properties. Although the addition of ZS masterbatch as the only addition in PE film does not significantly affect most of the parameters tested, its use in conjunction with ZO masterbatch significantly increases the antimicrobial activity of films against *E. coli* and *S. aureus* strains. More than it would appear from the sum of the activities of each of the add-ons. The synergy effect may be caused by better distribution of zinc at the interface due to the use of zinc stearate and several mechanisms affecting the increase in disturbance of the cell membrane potential. The function of microbiological activity against Gram (+) and Gram (-) bacteria and the acceptable level of migration indicate the universality of the presented solution and the possibility of using PE films containing nanoparticles of zinc oxide and zinc stearate for packaging various types of food products. Especially that the samples were carried out in industrial conditions.

## Figures and Tables

**Table 1:** Composition of PE films containing functional zinc compounds.

Sample	Control	ZO3	ZS3	ZO3/ZS3	ZO6	ZS6	ZO6/ZS6
Component	Concentration [% *w/w*]						
LDPE	58	55	55	52	52	52	46
mLLDPE	35						
CSE	1						
Plastomer	5						
Antistatic agent	1						
Zinc oxide*	**0**	**3**	**0**	**3**	**6**	**0**	**6**
Zinc stearate*	**0**	**0**	**3**	**3**	**0**	**6**	**6**

* In the form of a masterbatch prepared according to 2.2.1.

**Table 2:** Comparison of the optical properties of PE films containing a masterbatch with zinc oxide (ZO) and/or zinc stearate (ZS) (*n* = 3, *p* < 0.05).

**Sample**	**Transmittance at 280 nm [%]**	**Transmittance at 660 nm [%]**
C	82.4 ± 1.3 ^a^	58.6 ± 2.1 ^a^
ZO3	32.8 ± 0.9 ^b^	43.5 ± 1.2 ^b^
ZS3	83.9 ± 1.8 ^a^	59.8 ± 1.6 ^a^
ZO3/ZS3	29.6 ± 2.3 ^b^	44.0 ± 3.0 ^b^
ZO6	10.3 ± 0.6 ^c^	2.1 ± 0.2 ^c^
ZS6	85.2 ± 2.5 ^a^	60.5 ± 2.4 ^a^
ZO6/ZS6	10.5 ± 1.0 ^c^	3.6 ± 0.3 ^d^

Values with different letters marked from a to d differ significantly against the control sample in each column.

**Table 3:** Comparison the results of global migration of PE films containing a masterbatch with zinc oxide (ZO) and/or zinc stearate (ZS) (*n* = 3, *p* < 0.05).

Type of Model Fluid	Global Migration [mg/dm^2^]			
	H_2_O	3% (*w/v*) CH_3_COOH	10% (*v/v* EtOH	*95% (*v/v*) EtOH
Control	0.046 ± 0.002 ^a^	0.063 ± 0.003 ^a^	0.0019 ± 0.0002 ^a^	0.0010 ± 0.0002 ^a^
ZO3	0.088 ± 0.003 ^b^	0.588 ± 0.007 ^b^	0.0025 ± 0.0003 ^a^	0.0015 ± 0.0003 ^a^
ZS3	0.041 ± 0.004 ^a^	0.124 ± 0.005 ^c^	0.0020 ± 0.0003 ^a^	0.0014 ± 0.0003 ^a^
ZO3/ZS3	0.091 ± 0.004 ^b^	0.692 ± 0.010 ^d^	0.0028 ± 0.0005 ^a^	0.0018 ± 0.0003 ^a^
ZO6	0.155 ± 0.005 ^c^	1.230 ± 0.013 ^e^	0.0045 ± 0.0003 ^b^	0.0017 ± 0.0003 ^a^
ZS6	0.050 ± 0.004 ^a^	0.332 ± 0.009 ^f^	0.0025 ± 0.0003 ^a^	0.0015 ± 0.0002 ^a^
ZO6/ZS6	0.168 ± 0.009 ^c^	1.794 ± 0.013 ^g^	0.0051 ± 0.0004 ^b^	0.0020 ± 0.0005 ^a^

* Substitute for a hydrophobic model liquid. Values with different letters marked from a to g differ significantly against the control sample in each column.

**Table 4:** Comparison the mechanical properties of PE films containing containing a masterbatch with zinc oxide (ZO) and/or zinc stearate (ZS) (*n* = 10, *p* < 0.05).

Sample	Tensile Strength [MPa]	Young’s Modulus [MPa]	Elongation at Break [%]
Control	20.2 ± 0.6 ^a^	236.4 ± 2.3 ^a^	293 ± 18 ^a^
ZO3	24.9 ± 0.4 ^b^	250.5 ± 3.0 ^b^	256 ± 27 ^a^
ZS3	20.5 ± 1.0 ^a^	262.1 ± 1.3 ^c^	274 ± 15 ^a^
ZO3/ZS3	24.0 ± 0.5 ^b^	272.9 ± 2.5 ^d^	245 ± 19 ^b^
ZO6	25.2 ± 0.7 ^b^	283.6 ± 1.7 ^e^	189 ± 16 ^c^
ZS6	20.9 ± 0.6 ^a^	265.5 ± 3.3 ^c^	180 ± 20 ^c^
ZO6/ZS6	23.9 ± 0.7 ^b^	312.8 ± 3.7 ^f^	169 ± 29 ^c^

Values with different letters marked from a to f differ significantly against the control sample in each column.
